# A single-institutional review of 68 patients with dermatofibrosarcoma protuberans: wide re-excision after inadequate previous surgery results in a high rate of local control

**DOI:** 10.1186/s12957-016-1075-2

**Published:** 2017-01-05

**Authors:** Kamran Harati, Kim Lange, Ole Goertz, Armin Lahmer, Nicolai Kapalschinski, Ingo Stricker, Marcus Lehnhardt, Adrien Daigeler

**Affiliations:** 1 Department of Plastic Surgery, Burn Centre, Hand Centre, BG-University Hospital Bergmannsheil, Buerkle-de-la-Camp-Platz 1, 44789 Bochum, Germany; 2 Institute of Pathology, Ruhr-University Bochum, Buerkle-de-la-Camp-Platz 1, 44789 Bochum, Germany

**Keywords:** Dermatofibrosarcoma, Recurrence, Survival, Margin, Excision

## Abstract

**Background:**

Dermatofibrosarcoma protuberans (DFSP) is a cutaneous soft tissue sarcoma characterized by an indolent but aggressive local growth. Unplanned excisions with positive margins are common, and the prognostic impact of radical re-excisions is still unclear. The aim of the present study was to identify prognostic indicators of recurrence-free survival (RFS) in patients with DFSP through a long-term follow-up. We tried particularly to determine the prognostic impact of surgical margins and re-excisions in patients after earlier inadequate surgery.

**Methods:**

Seventy-five patients with DFSP were treated surgically at our institution between 1999 and 2015. Analyses were restricted to 68 participants with available information on surgical margins. The median follow-up was 5.4 years.

**Results:**

Fifty-four patients (79.4%) had low-grade DFSP and 14 patients (20.6%) intermediate-grade FS-DFSP. The 5-year RFS rates were estimated to be 93.5% (95% CI 81.2–97.9) for low-grade DFSP and 39.7% (95% CI 13.0–65.8) for FS-DFSP (*P* < 0.0001). Re-excisions were performed in 55 patients (80.9%) following R1 or marginal R0 resections. Negative margins could be attained in a total of 65 patients (95.6%). Negative margin widths >1 cm led to the best local outcome within the R0 subgroup. Significant adverse prognostic features in the multivariate analysis included histologic grade and close margins.

**Conclusions:**

The data from this study underscore the long-term benefit of negative margins. In our analysis, re-excisions were an effective method to achieve a high rate of local control in patients who presented after R1 or marginal R0 resection. To ensure the best outcome, re-excisions should aim at negative margin widths of more than 1 cm in the histologic specimen.

## Background

Dermatofibrosarcoma protuberans (DFSP) is a rare, cutaneous soft tissue sarcoma of fibroblastic origin. It accounts for approximately 1% of all soft tissue sarcomas with an estimated incidence of three to five cases per million persons [1, 2]. DFSP can occur throughout the body, but it is localized predominantly at the trunk and the proximal extremities [3, 4]. Its clinical behaviour is characterized by an indolent, but local, aggressive growth with destructive infiltration of the surrounding tissues. Patients rarely die from DFSP due to its low metastatic potential: less than 5% of all patients develop distant metastases [4–8]. Histologically, approximately 90% of all DFSPs are low-grade lesions while 10% contain fibrosarcomatous components (FS-DFSP). The FS-DFSP subtype is, therefore, considered as an intermediate-grade sarcoma displaying a more aggressive growth pattern with a higher rate of local recurrences and metastases [9–12].

At a molecular level, more than 90% of all DFSP arise from the translocation of chromosomes 17 and 22, resulting in a fusion between the collagen type Iα1 gene (COL1A1) and the platelet-derived growth factor β-chain gene (PDGFB) [13, 14]. This rearrangement causes a continuous activation of platelet-derived growth factor receptor β (PDGFRβ) protein tyrosine kinase, which promotes DFSP cell growth. The correlating signalling pathway can be targeted by tyrosine kinase inhibitors, such as imatinib and sunitinib, which have been revealed to be promising candidates in the treatment of metastatic or locally advanced DFSP that were not suitable for further curative resection [15–17]. Currently, imatinib is approved in Europe for the treatment of inoperable tumours and metastatic DFSP.

The standard curative treatment for DFSP to date still remains surgical resection with wide, clear margins. Due to its distinctive storiform growth pattern with pseudopod-like extensions, incomplete excisions are relatively common, especially in “whoops” procedures, where banal lesions were expected preoperatively. The data available on local recurrence rates after surgical excision are relatively heterogeneous, ranging from 0 to 57% [6, 8, 18]. Notably, the local recurrence rates for Mohs micrographic surgery (MMS) reported, ranging from 0 to 1.1%, are significantly superior to those for wide local excisions (WLE), at 0 to 27% [2, 3, 5, 7, 8, 19–27]. The European Association of Dermato-Oncology (EADO) and the European Organization of Research and Treatment of Cancer (EORTC) recommend MMS to reduce the local recurrence rates and to minimize the amount of excised tissue, particularly at the head and neck area, in a position paper published currently. However, WLE with a lateral safety margin of 3 cm was advised in treatment centres where only standard histopathological procedures are available [28]. Several treatment centres suggested safety margins ranging from 2 to even 5 cm, but they did not differentiate clearly between the safety margin planned preoperatively and the margin width histologically assessed postoperatively [2, 7, 8, 10, 24, 25, 27, 29]. Nevertheless, it seems reasonable that wider safety margins might be associated with more favourable outcomes, but a more precise cutoff point has yet to be assessed. Although reconstructive plastic surgery can frequently reduce functional impairment and cover soft tissue defects, the surgical approach with such wide safety margins is associated with considerable morbidity and should, therefore, be weighed up carefully.

In the current study, we reviewed our own institutional experience and assessed the prognostic significance of negative margin widths in patients with low-grade DFSP and FS-DFSP. In particular, we tried to determine the prognostic impact of re-excisions in patients after inadequate surgery.

## Methods

### Patients

Seventy-five patients with DFSP of the trunk, the extremities, the head and the neck were treated surgically at our institution between January 1999 and September 2015. A total of 20 of the 75 patients presented with primary disease at our institution. Fifty-five patients were referred to our tertiary centre subsequently after incomplete resections. From this group, we excluded five patients because essential data regarding the initial surgical procedure, such as tumour size or margin status, were not available. Furthermore, two patients were lost to follow-up. Thus, we restricted the analyses to 68 participants with full information available on the outcome and surgical margins at the initial procedure. They were assessed and their clinicopathological characteristics are summarized in Table 1. Patient follow-up was obtained from our database, medical records and patient correspondence.

**Table 1:** Results of univariate analyses to determine patient and tumour dependent factors predictive of recurrence-free survival

	*N*	Estimated 1-year RFS (95% CI)	Estimated 2-year RFS (95% CI)	Estimated 5-year RFS (95% CI)	*P* (log-rank)*
Age (years)					
<60	55	96.2 (85.6–99.0)	91.5 (78.7–96.7)	86.5 (72.3–93.8)	0.075
≥60	13	69.2 (37.3–87.2)	69.2 (37.3–87.2)	69.2 (37.3–87.2)	
Sex					
Female	35	81.8 (63.9–91.4)	78.4 (59.9–89.1)	78.4 (59.9–89.1)	0.237
Male	33	100 (−)	96.2 (75.7–99.4)	87.2 (65.2–95.7)	
Site					
Upper extremity	20	77.1 (49.5–90.9)	70.7 (42.8–86.8)	63.6 (35.7–82.0)	0.013
Lower extremity	17	93.8 (63.2–99.1)	93.8 (63.2–99.1)	93.8 (63.2–99.1)	0.237
Head/neck	8	85.7 (33.4–97.9)	85.7 (33.4–97.9)	71.4 (25.8–92.0)	–
Trunk	23	100 (−)	94.1 (65.0–99.1)	94.1 (65.0–99.1)	0.086
Tumour size					
<2 cm	36	94.3 (79.0–98.5)	90.8 (73.9–97.0)	90.8 (73.9–97.0)	0.123
≥2 cm	32	86.1 (67.1–94.6)	82.4 (62.7–92.3)	73.5 (51.8–86.5)	
Histologic grade					
G1 (low-grade DFSP)	54	95.9 (84.7–99.0)	93.5 (81.2–97.9)	93.5 (81.2–97.9)	
G2 (FS-DFSP)	14	68.1 (35.4–86.8)	59.6 (28.2–80.9)	39.7 (13.0–65.8)	<0.0001

*RFS* recurrence-free survival, *CI* confidence interval

*Log-rank test for equality of survivor functions

### Ethics approval and consent to participate

The retrospective analysis was approved by the Ethics Committee of the Medical Faculty of the Ruhr-University Bochum (Registration number 15-5441). Patients gave written informed consent to participate.

### Treatment

The goal of surgical treatment for all patients was the complete resection of the primary or residual tumour with wide, clear margins. This resection included the scars from previous surgeries, biopsies and wound drainage. If necessary, immediate or delayed soft tissue coverage was performed with split thickness skin grafts, and local or free flaps. No patients in this series were treated with MMS.

The indication for adjuvant radiation was given at the discretion of the interdisciplinary tumour board of our institution. Adjuvant radiation was recommended after incomplete surgical resection.

### Histopathological classification

All tumours were diagnosed and classified using the guidelines of the French Federation of Cancer Centres (FNCLCC) and those of the World Health Organization (WHO). Surgical margins were assessed after fixation of the pathologic specimen with formalin and dyeing the surface with ink. All pathology slides were analysed or reviewed for consensus diagnosis by experienced soft tissue pathologists from our institution.

### Statistical analysis

All patients were retrospectively analysed regarding possible prognostic factors influencing recurrence-free survival (RFS). The RFS was defined as the time period from the date of surgery at our institution to the date of first recurrence or censored at the date of last follow-up assessment in recurrence-free patients. The RFS rates were estimated according to the Kaplan-Meier method with respective 95% confidence intervals (CIs) and were compared using the log-rank test. Multivariate analyses were performed using the Cox proportional hazards model and the Wald test. Variables that were associated with *P* < 0.05 in the univariate analysis were included in the multivariate regression to assess independent prognostic factors. The data analysis was performed using the statistical programme Stata (Version 11.2, StataCorp, College Station, TX, USA).

## Results

### Patient characteristics and surgical margins

The median follow-up after primary diagnosis as of February 2016 (cutoff date) was 5.4 years. The median age at the time of primary diagnosis was 42.2 years (range 2.9–75.6). There were 35 female and 33 male patients. Fifty-four patients (79.4%) had low-grade (G1) DFSP and 14 (20.6%) had intermediate-grade (G2) FS-DFSP. The tumours were located in the lower extremities in 17 patients (25%) in the upper extremities in 20 patients (29.4%), in the trunk in 23 patients (33.9%) and in the head and neck area in eight patients (11.8%). Sixty-two patients had epifascial tumours, while only six patients had tumours with subfascial involvement. The median tumour size was 1.9 cm (range 0.1–20.0 cm).

The primary resection at the initial surgical procedure led to microscopically negative margins (R0) in only 13 patients (19.1%), whereas 54 patients (79.4%) were left with microscopically positive margins (R1) and one (1.5%) with macroscopically positive margins (R2). Three patients with FS-DFSP received adjuvant radiotherapy after the resection of the primary tumour. Nine more patients underwent radiotherapy after initial local recurrence.

Fifty-three patients with incompletely resected tumours and two patients after marginal R0 resection subsequently underwent re-excisions at our institution. Residual disease was detected histologically in the pathologic specimen in 47 of these 55 patients (85.5%). At the re-excision, microscopically negative margins were attained in 52 of the 55 patients mentioned (94.5%). Tumours infiltrated critical anatomic structures or were too advanced and widespread for complete resection that would have resulted in function loss and increased morbidity in the remaining three patients with R1 margins. Hence, microscopically positive margins were tolerated consensually in these patients. Continuous follow-ups with contrast-enhanced MRIs detected progressive disease in two of these patients. The histologically assessed closest negative margin width could be obtained from the database in 48 patients with R0 resected tumours at the very initial procedure or forthcoming re-excisions. Plastic surgical soft tissue coverage had to be performed in 32 patients (47.1%) involving local flaps (28), free flaps (3) and split thickness skin grafting (1). One patient was amputated at the proximal phalanx of his ring finger after early local recurrence.

Tumour resection and soft tissue coverage were usually performed as a one-step procedure to minimize the risk of wound contamination or infection. However, in some cases where complicated flaps were required for soft tissue reconstruction, the defects were covered temporarily with vacuum dressings. Six (21.4%) of the local flaps and one (33.2%) of the free flaps had been transferred in a second or even third procedure after histological examination.

A lateral clear margin of 2.0 cm of normal tissue was intended wherever feasible. The median negative margin width was 0.35 cm in patients presenting with primary tumours at our institution. Patients that underwent incomplete resections at the referring institutions or practices were resected with a gross margin width of 2.0 cm around the scars. The median clear margin width was 0.8 cm in re-excised patients.

A total of ten patients had at least one local recurrence during the follow-up period, whereas four patients had two or more local recurrences (range 2–6). The tumour and treatment characteristics of these ten patients are displayed in Table 2. No patient developed distant metastases or died because of disease, while three patients died for reasons other than DFSP.

**Table 2:** Tumour and treatment characteristics of patients with local recurrence

Case no.	Entity	Number of recurrences	Margin status after initial resection of the primary tumour	Re-excision	Definite margin status	Negative surgical margin width (cm)	Time to first recurrence (months)
1	DFSP	2	R0	No	R0	0.3	24
2	FS-DFSP	1	R1	No	R1	–	26
3	DFSP	1	R0	No	R0	0.2	4
4	FS-DFSP	1	R0	No	R0	0.2	14
5	FS-DFSP	1	R1	Yes	R0	0.3	4
6	FS-DFSP	8	R1	Yes	R0	1.0	9
7	FS-DFSP	6	R1	Yes	R1	–	30
8	FS-DFSP	5	R1	Yes	R0	0.8	8
9	FS-DFSP	1	R1	Yes	R0	0.2	3
10	DFSP	1	R0	No	R0	Not available	5

*RFS* recurrence-free survival, *CI* confidence interval

### Univariate analysis of survival

**Fig. 1:**
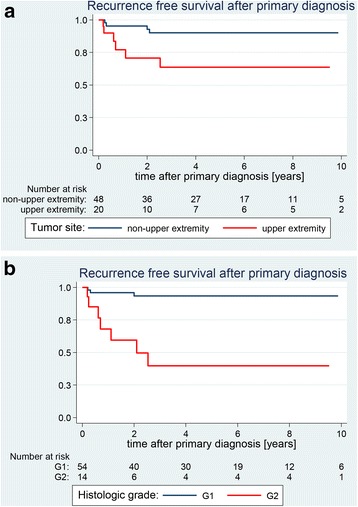
Estimated recurrence-free survival curves depending on tumour site (**a**) and histologic grade (**b**)

The Kaplan-Meier estimated rate of RFS after 5 years was 82.6% (95% CI 70.0–90.3) for the entire series. Age, gender and tumour size were not found to be significant predictors of RFS (Table 1). Tumours arising in the upper extremities had a worse prognosis compared to lesions at other sites (5-year RFS 63.6% [35.7–82.0] vs. 90.1% [75.6–96.2]; *P* = 0.013) (Fig. 1a). Similar to findings in other studies, patients with low-grade (G1) DFSP had more favourable prognoses than patients with intermediate (G2) FS-DFSP. The 5-year RFS rates were estimated to be 93.5% (95% CI 81.2–97.9) for G1 DFSP and 39.7% (95% CI 13.0–65.8) for G2 FS-DFSP (*P* < 0.0001) (Table 1, Fig. 1b).

**Fig. 2:**
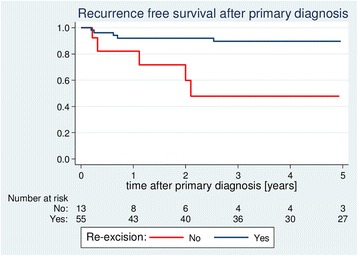
Effects of re-excision on recurrence-free survival

It is noteworthy that patients who had already undergone a R0 resection at the initial procedure appeared to have a worse prognosis compared to patients who had been resected only incompletely and, therefore, had to undergo further re-excision (5-year RFS 63.3% [28.6–84.6] vs. 87.1% [73.3–94.0]), although this survival distribution failed to reach statistical significance in the univariate analysis and a borderline *P* value was attained (*P* = 0.053) (Table 3). However, patients who underwent re-excision had a significantly improved RFS (5-year RFS 89.6% [76.7–95.6] vs. 47.9% [15.5–74.7]; *P* = 0.002) (Fig. 2).

**Table 3:** Univariate analyses of recurrence-free survival depending on treatment characteristics

	*N*	Estimated 1-year RFS (95% CI)	Estimated 2-year RFS (95% CI)	Estimated 5-year RFS (95% CI)	*P* (log-rank)*
Margin status after initial resection					
R0	13	83.1 (47.2–95.5)	63.3 (28.6–84.6)	63.3 (28.6–84.6)	
R1/2	55	91.9 (79.8–96.9)	91.9 (79.8–96.9)	87.1 (73.3–94.0)	0.053
Re-excision					
No	13	82.1 (44.4–95.3)	59.8 (24.1–83.1)	47.9 (15.5–74.7)	
Yes	55	92.0 (80.1–96.9)	92.0 (80.1–96.9)	89.6 (76.7–95.6)	0.002
Margin status after first treatment round (initial resection + re-excision)					
R0	65	89.9 (78.8–95.3)	86.0 (73.9–92.8)	84.0 (71.3–91.4)	
R1	3	100 (−)	100 (−)	(−)	–
Wound closure during first treatment round					
Primary closure	35	80.8 (62.1–90.9)	73.6 (53.8–85.9)	69.7 (49.6–83.1)	
Non-primary closure (plastic surgical tissue transfer)	32	100 (−)	100 (−)	96.2 (75.7–99.4)	0.006
Adjuvant radiotherapy					
No	65	89.9 (78.8–95.3)	86.0 (73.9–92.8)	82.0 (69.0–89.9)	
Yes	3	100 (−)	100 (−)	100 (−)	–
Distance of closest negative margin (R0 group) in cm					
≤0.2	11	81.8 (44.7–95.1)	71.6 (35.0–89.9)	71.6 (35.0–89.9)	
0.2–0.5	11	88.9 (43.3–98.4)	71.1 (23.3–92.3)	71.1 (23.3–92.3)	
0.5–1.0	10	77.8 (36.5–93.9)	77.8 (36.5–93.9)	77.8 (36.5–93.9)	
>1.0	16	100 (−)	100 (−)	100 (−)	0.039**

*RFS* recurrence-free survival, *CI* confidence interval

*Log-rank test for equality of survivor functions, **Global log-rank test for the trend of survivor functions

Margins above 1 cm in the univariate analysis of categorized clear margin widths led to a significantly better outcome when compared with closer margins (Table 3). Another interesting observation was made when analysing the type of wound closure carried out after the first treatment round. Here, the use of reconstructive plastic surgical soft tissue coverage involving skin grafting and local and free flaps was associated with a more favourable outcome when compared to primary closure (5-year RFS 96.2% [75.7–99.4] vs. 69.7% [49.6–83.1]; *P* = 0.006) (Table 3).

### Multivariate analysis of survival

The most significant prognostic factor for RFS in the Cox model was the histologic grade (Table 4). The hazard ratio (HR) for local recurrence was 5.99 (95% CI 1.15–31.34; *P* = 0.034) for patients with G2 FS-DFPS. Re-excision was another independent predictor of RFS: Patients who underwent re-excision had a significantly lower risk of local failure (HR 0.23; 95% CI 0.06–0.93; *P* = 0.039). The site of the tumour and the use of reconstructive soft tissue coverage were significant indicators of RFS in the univariate analysis but failed to reach statistical significance in the multivariate analysis.

**Table 4:** Results of multivariate analysis on recurrence-free survival in the entire cohort (*N* = 68)

Category (reference)	Hazard ratio	95% CI	*P*
Histologic grade: G2 (vs. G1)	5.99	1.15–31.34	0.034
Tumour site: upper extremity (vs. other)	2.21	0.43–11.28	0.339
Wound closure at primary resection: primary (vs. tissue transfer)	6.48	0.76–55.38	0.088
Re-excision: yes (vs. no)	0.23	0.06–0.93	0.039

*CI* confidence interval

In an additional multivariate analysis, we determined independent prognostic factors for RFS in the subgroup of patients with R0 resections and data available on margin widths (Table 5). In accordance with the multivariate analysis of the entire cohort, the histologic grade was found to be an independent predictive factor. Moreover, a negative margin width >1.0 cm could also be determined as a significant independent prognostic factor of RFS.

**Table 5:** Results of multivariate analysis on recurrence-free survival in the subgroup of patients with data available on negative margin widths (*N* = 48)

Category (reference)	Hazard ratio	95% CI	*P*
Histologic grade: G2 (vs. G1)	9.43	2.38–37.43	0.001
Tumour site: upper extremity (vs. other)	1.60	0.7–38.4	0.763
Wound closure at primary resection: primary (vs. tissue transfer)	5.84	0.68–50.18	0.108
Closest negative margin width: >1.0 cm (vs. ≤1.0 cm)	0.01	0.01–0.03	<0.001

*CI* confidence interval

### Regression analysis of non-categorized surgical margin width

Clear margins were found to be a significant predictor of RFS in the Cox regression analysis and were inversely proportional to the risk of local recurrence: the wider the negative margin width, the lower the risk of local failure. This distribution in the Wald test was statistically significant, and the HR for local recurrence was 0.22 (95% CI 0.06–0.80) for negative margins with >1 cm healthy tissue at the closest distance (*P* = 0.021).

## Discussion

The rarity of DFSP and the low number of recurrence events pose epidemiological challenges and preclude even large studies to assess the prognostic factors of RFS. Dermatofibrosarcoma protuberans can lead to significant morbidity due to its aggressive local growth and the high rates of local recurrence despite surgical resection. In the present study, 15.4% of all R0 resected patients developed a local recurrence during the course of the disease. The two largest, well-characterized studies from the Instituto Nazionale Tumori (INT) and the Memorial Sloan-Kettering Cancer Center (MSKCC) presented cohorts with local recurrence rates of 4.3 and 22.1%, respectively [4, 5]. The distinct variation between those two studies reflects the differences in tumour histology, as the INT series contained a lower percentage (3.2%) of patients with the more aggressive FS-DFSP subtype than the MSKCC series (17%). In the present study, a relatively high proportion of patients (20.6%) had a FS-DFSP, which was found to be one of the most significant adverse predictors of RFS in our study. Hence, the observation of high recurrence rates is probably due to differences in patient selection and the corresponding tumour pathology between the two institutions reported and our institution.

Incomplete resections in the form of “whoops” procedures are common in the previous history of DFSP patients. A total of 79.2% of all patients referred came to our institution after incomplete resections. However, further re-excision led to negative margins in nearly all cases, except for three patients. It is notable that pathologic residual disease could be detected in 85.5% of the re-excised patients. In our series, re-excision to achieve wide, clear margins has been determined to be an effective method of achieving a high rate of local control in patients who presented after R1 or marginal R0 resection. This finding provides a liberal policy of re-excision after inadequate surgery.

It is interesting that re-excision appeared to be a significant predictor of RFS when analysed as a potential prognostic event. The reason, therefore, might lie in the fact that re-excision led to wider negative margins. The margin width attained surgically was a statistically significant predictor of the outcome in the univariate and the regression analysis in the subgroup analysis of 48 patients with negative margins. None of the patients with negative margins above 1 cm had a local recurrence during follow-up. Accordingly, a surgical margin width of 1 cm was a cutoff point in our series. However, this was the histologically assessed margin width in the pathologic specimen, and we cannot conclude which safety margins planned preoperatively are needed in order to obtain a histologically clear margin of 1 cm. Hence, the recommended lateral safety margins ranging from 2 to 3 cm might be justified in most of the cases localized at the trunk or proximal extremities, whenever feasible [7, 10, 23, 27, 28].

A high need for reconstructive procedures was observed in our patient population (47.1%) as a consequence of wide and radical re-excisions. Reconstructive procedures had to be performed in 30.2, 30.3 and 72.2% of all patients treated in other institutions that did not use MMS [3, 5, 8]. It is notable that the highest rate was reported by a plastic surgical department, presumably due to a selection bias. This observation might also give the impression that plastic surgeons are more inclined to perform reconstructive procedures when they are also responsible for tumour resection. However, these retrospective observations cannot be used to evaluate the treatment approaches of the different disciplines involved. The use of reconstructive surgery in our series displayed an improved outcome in univariate analysis, but this distribution failed to reach statistical significance in multivariate analysis, because the use of reconstructive surgery was associated with a larger margin width. The median negative surgical margin width was 0.5 cm for primary closed wounds and 1.3 cm for wounds with plastic surgical tissue transfer. The reason, therefore, might be a selection bias where wider resections necessitated the use of reconstructive surgery more frequently. Vice versa, plastic surgical tissue transfer enabled wider resections due to the safe wound coverage and, therefore, might have led to more radical resections.

Only three patients received adjuvant radiation in our series. Thus, we cannot make any reliable conclusions regarding the efficacy of adjuvant radiotherapy. Nevertheless, adjuvant radiotherapy remains a treatment option for tumours that only can be resected with close or positive surgical margins in critical anatomic sites [5, 30]. Radiation can also be used as an exclusive treatment option in locally advanced tumours where further surgery is not possible [31]. A retrospective study by the MD Anderson Cancer Center analysed the outcome of 53 DFSP patients treated with pre- or postoperative radiation and presented a local control rate of 98% after 5 years [32]. The authors recommended adjuvant radiation for patients with large or recurrent tumours or when surgical attempts at wide margins would result in significant morbidity. Finally, the question remains whether adjuvant radiotherapy should be applied in patients with FS-DFSP despite negative margins. To date, there are no studies that have analysed the prognostic effects of radiation on both DFSP subtypes separately. Hence, there are no clear suggestions whether radiation should be applied generally in patients with R0-resected FS-DFSP.

There are no randomized controlled studies to date comparing MMS and WLE, but MMS seems to result in significantly lower local recurrence rates [3, 19–22]. Mohs micrographic surgery should be preferred particularly in the treatment of head and neck lesions, because it allows greater preservation of normal tissue, resulting in a better functional and aesthetic outcome [33]. Although superior to WLE, MMS has some limitations when large or subfascial tumours at the trunk or the distal extremities are involved [3]. However, the current series is not able to comment on the use of MMS, because there were no patients in our database who were treated with MMS, either in our or the referring institutions. Despite consistent reports about the advantages of MMS, it is still not a common procedure in German clinical routine [15].

Finally, the intervals of follow-up care should be addressed so that recurrences can be detected at an early disease stage. Unfortunately, the other centres did not delineate their follow-up strategies. The German guidelines recommend clinical examinations only every 6 months during the first 5 years but state explicitly that reliable data do not exist [34]. In the present study, the mean time to local recurrence was 9 months in patients in whom local recurrence occurred. However, we were not able to assess the median time to local recurrence because of the low number of recurrence events. The recent MSKCC series with 159 patients reported a medium time to local recurrence of 32 months [6]. Therefore, intense follow-ups during the first 5 years seem reasonable. The last recurrence in our series occurred after two and a half years. This observation raises the question whether a follow-up up to 5 years might be adequate for patients with DFSP or should continue even after 5 years. In contrast to our study, the INT and MSKCC also reported late recurrences that developed more than 5 years after primary diagnosis. However, because of the small number of events after 5 years, we cannot conclude whether follow-up examinations are necessary after this long time period. The follow-up management for DFSP in our institution includes clinical examinations and contrast-enhanced MRIs every 3 months in the first 2 years and then every 6 months for three more years. The decision whether follow-up MRIs should be continued after 5 years for every 6 or 12 months is based on the previous tumour behaviour and the decision of the informed patient. Finally, we have to state that this follow-up strategy is only based on our own experiences with a small proportion of patients suffering late recurrences and is not supported by any data of high evidence.

## Conclusions

In summary, this study provides long-term follow-up data that may help clinicians estimate the prognosis of patients with DFSP more accurately and to guide clinical decisions after inadequate surgery and when MMS is not available. Adverse prognostic features in our series included the FS-DFSP subtype and close resection margins. The data from this study could underscore the benefit of re-excision after R1 and marginal R0 resection. Re-excision to attain wide, clear margins has been determined to be an effect method to achieve a high rate of local control in the large subset of patients who present after R1 or marginal R0 resection.

## Abbreviations

CI: Confidence interval
DFSP: Dermatofibrosarcoma protuberans
FS-DFSP: Fibrosarcomatous dermatofibrosarcoma protuberans
MMS: Mohs micrographic surgery
RFS: Recurrence-free survival
WLE: Wide local excision
